# Mean Evan's Index among Patients with Normal Computed Tomography Scan visiting Radiology Department in a Tertiary Care Centre of Nepal: A Descriptive Cross-sectional Study

**DOI:** 10.31729/jnma.7006

**Published:** 2021-08-31

**Authors:** Sagar Koirala, Prity Agrawal, Suresh Bishokarma, Pratyush Shrestha

**Affiliations:** 1 Department of Neurosurgery, Upendra Devkota Memorial Neurological and Allied Sciences, Bansbari, Kathmandu, Nepal; 2 Department of Radiology, Upendra Devkota Memorial Neurological and Allied Sciences, Bansbari, Kathmandu, Nepal

**Keywords:** *computed tomography*, *Evan's index*, *hydrocephalus*

## Abstract

**Introduction:**

Evan's index is useful to objectively see if ventricles size is abnormal especially in borderline cases of hydrocephalus. Studying ventricular size in CT scan is essential in every pathology of the brain. Use of objective parameters to define hydrocephalus helps us not only to diagnose a case but also follow up the case following treatment. The aim of this study was to find out the mean evan index among patients visiting the department of radiology of a tertiary care hospital.

**Methods:**

A descriptive cross-sectional study was conducted at a tertiary care hospital from 1st january 2020 to 31st December 2020. Ethical clearance was obtained from the Institutional Review Committee of Upendra Devkota Memorial Neurological and Allied Sciences (reference number: 116/2021). Computed tomography scans were done for various reasons in the hospital over a one year period and reported normal by the radiologists were included in the study. Convenient sampling was done. Statistical analysis was done using Statistical Package for the Social Sciences. Point estimate at 95% Confidence Interval was calculated along with mean and standard deviation for continuous data.

**Results:**

In this study, among the 216 cases, the mean Evan's index was found to be 0.20±0.04.

**Conclusions:**

The mean evan's index in our study population was lower than the normal cut-off value.

## INTRODUCTION

Study of ventricular size in CT scan is essential in every pathology of the brain.^1^ There are various parameters used to diagnose enlargement of ventricles. Subjective parameters like mickey mouse sign are easy to identify in overtly dilated ventricles.^2^

Use of objective parameters to define hydrocephalus helps us not only to diagnose a case but also follow up the case following treatment. Evan's index is defined as the ratio between maximum bifrontal horn width and largest biparietal diameter.^3^ In normal people this ratio is less than 0.3.^3^ The frontal horn width ratio (FHWR) is also commonly used and is defined as maximum bifrontal horn width to internal frontal diameter at the same level where bifrontal horn width is calculated.^4^ This ratio is less than 0.5 normally.^4^

The aim of this study was to find out the mean Evan index among patients visiting the department of radiology of a tertiary care hospital.

## METHODS

A descriptive cross-sectional study was conducted at Upendra Devkota Memorial National Institute of Neurological and Allied Sciences from 1st January 2020 to 31st December 2020 after clearance by institutional review committee (ref no. 116/2021). All CT scans which were done for various reasons in the hospital and reported normal by the radiologists were included in the study. All other CT scans which had abnormal radiological findings were excluded from the study. Convenient Sampling method was used. Sample size was calculated using the formula,

n = Z^2^ × d^2^ / e^2^

  = (1.96)^2^ × (0.04)^2^ / (0.01)^2^

  = 105

Where,

n = minimum required sample size,
Z = 1.96 at 95% Confidence Interval (CI)
d = Standard deviation taken from a previous study
e = margin of error, 1%

Since the convenient Sampling method was used, doubling the calculated sample size, it becomes 210. Therefore the sample size was 210.

All the morphometric measurements were taken by the radiologist in the consul of CT scanner. Evan's index which is the ratio between maximum bifrontal horn width and maximum biparietal diameter was calculated. Mean and standard deviations were calculated from overall data as well as among sex distribution and age distribution. Statistical analysis was done using Statistical Package for the Social Sciences version 20. Point estimate at 95% Confidence Interval was calculated along with mean and standard deviation for continuous data.

## RESULTS

Out of 216 cases, the mean Evan's index was found to be 0.20±0.04. A total of 216 cases were included in the study out of which either sex was 108 (50%). The youngest case was 2 years of age and the oldest 87 with a mean of 34.5±16.9 years. Mean biparietal diameter was 127.18±6.55 and mean frontal horn width was 26.03±5.51.

Overall, the mean Evan index was less in females than in males. However, both the groups have these ratios below the normal cutoff of 0.03 (Table 1).

**Table 1:** Sex wise distribution of Evan's Index.

Indices	Sex	n (%)	Mean±SD
Evan's Index	Male	108 (50)	0.21±0.04001
	Female	108 (50)	0.19±0.04106
Biparietal diameter	Male	108 (50)	129.13±6.4503
	Female	108 (50)	125.23±6.0843
Frontal horn width	Male	108 (50)	27.58±5.2042
	Female	108 (50)	24.47±5.4040

Mean Evan's index among all age groups were within the normal limit of 0.3 (Table 2).

**Table 2:** Age wise Mean Evan's ratio and its associated parameters.

Age Group	n (%)	Mean Evan's ratio	Mean biparietal diameter (mm)	Mean frontal horn width (mm)
0-10	19(8.79)	0.16±0.03	129.07±6.33	21.56±5.35
11-20	24 (11.11)	0.21±0.03	130.86±6.91	27.98±4.65
21-30	55 (25.46)	0.19±0.04	127.05±7.04	24.63±5.83
31-40	46 (21.29)	0.20±0.03	125.37±6.47	26.36±5.57
41-50	34 (15.74)	0.20±0.03	126.92±5.69	26.58±5.69
51-60	24 (11.11)	0.21±0.03	126.30±5.60	27.02±4.74
61-70	8 (3.7)	0.29±0.03	127.10±7.30	30.87±2.77
71-80	4 (1.85)	0.29±0.04	126.50±5.67	30.55±4.58
81-90	2 (0.92)	0.24±0.14	126.80±3.25	25.95±12.37

## DISCUSSION

Imaging plays a key role in diagnosis of hydrocephalus. Initially CT scan and now MRI are gold standard in diagnosis hydrocephalus.^5^ Various objective parameters have been described. Here we have used Evan's index and frontal horn width ratio in this study.

In our study we found that males have a slighter larger ventricular system in terms of Evan's index which came out to be statistically significant. These findings were also seen in studies done in other population.^3^ However the study in Ghanaians, Nigerians and South Indian population did not show any statistical difference.^6-8^ The mean values for both sexes are within the normal cutoff of 0.3 for Evan's index in our study.

In our study we did not find a linear increase in Evan's index with an increasing age group as in study of other populations. However, it holds valid in all age groups within the given cutoff in our population with normal CT scans.

Volumetric scans of ventricles and brain parenchyma are better than the linear indices in determining conditions like normal pressure hydrocephalus.^9^ However these linear indices are easier to calculate. Other linear indices like anterior posterior diameter of the lateral ventricles are also being studied to correlate with ventricular volumes.^10^

The other use of these indices is to objectify the ventricular size after treatment of hydrocephalus. Evan's index and FHWR has been used in various study to see the response to treatment after endoscopic third ventriculostomy.^11,12^ These indices also has been used in studies of response of lumboperitoneal and ventriculoperitoneal shunts.^13,14^ Knowing normal values of these indices is hence essential.

## CONCLUSIONS

The mean Evan's index was lower than normal in our study population.
